# Oral Pathology and Practice

**Published:** 1901-08

**Authors:** 


### Bibliography.

ORAL PATHOLOGY AND PRACTICE. A Text-Book for the Use of Students in Dental Colleges and a Hand-Book for Dental Practitioners. By W. C. Barrett, M.D., D.D.S., M.D.S., LL.D., Professor of the Principles and Practice of Dentistry and Oral Pathology in the University of Buffalo Dental Department; Professor of Dental Anatomy and Pathology in the Chicago College of Dental Surgery, etc. Second Edition. Revised, Enlarged, and Illustrated. The S. S. White Dental Manufacturing Company, Philadelphia, 1901.

The fact that in three years this work of the author, Dr. W. C. Barrett, has exhausted the first edition is sufficient evidence that it has filled a need in dental education.

The first edition was, in the opinion of the reviewer, very defective for several reasons, the main one being that of an entire absence of illustrations. These may be and generally are overdone, but a good cut, representing fully the ideas sought to be conveyed in the text, is an important auxilliary in education. The author acknowledges that he profited in the preparation of the second edition by the criticism made upon the first, and has very fully and, it is thought, judiciously illustrated the present volume.

The number of pages has been increased to three hundred and nineteen, "not a chapter reading as it did originally," the author states in his preface.

An effort seems to have been made to harmonize the author's views with those held by the dental profession, an effort not apparent in the first. This is especially true in regard to dentition, but even here there is still a tendency to what the reviewer would call false reasoning. This is manifest in the following paragraph:

"The condition of the gums should be carefully noted. If they are normal, without any special inflammation or thickening, we should look elsewhere for the source of the irritation."

This would lead any one following this advice into wrong conclusions, for the irritation from dentition very frequently is manifested long before resorption of the bony crypt takes place; indeed, frequently, as the reviewer has demonstrated, several years prior to eruption in the second permanent molar and proportionately so in all the developing teeth. If the practitioner waited for this indication upon the gums, an impacted third molar might send the patient to an insane asylum or an early grave before he would be able to diagnose the origin of the disturbance; but the author, apparently, does not regard permanent dentition of any importance, for it is not mentioned.

The author, when he does lance, regards the "crucial incision" as the best. A cut of this character is more difficult to make and is not as effective in releasing the cusps as that made in the form of the letter X.

The reviewer must agree with the author when, in discussing capping of the pulps, he writes, "When the pathological condition shall have proceeded to the extravasation into the body of the tissue of inflammatory products, there are practically no lymphatics to take them up, and their removal is as impossible as is that of any great effusion in the brain. Pulp-capping under such circumstances will be a hopeless proceeding, and the presence of any infiltrated or effused matter will contraindicate it." The pulp when free from inflammatory products is, in the opinion of the reviewer, a possible subject for capping.

The author's arrangement of the diseases of the pulp are far from satisfactory; indeed, he indulges in generalizations to an extent that must be confusing to the student. The reviewer would recommend to the author a perusal, if not adoption, of Arkövy on the various lesions of the pulp. He occupies one extreme, and our author the other. The reader will look in vain for any description of superficial pulpitis, a pathological condition long since recognized by writers in Europe as well as by some in this country.

The treatment of pericementitis as given is, in many respects, unphilosophical and almost borders on empiricism. Such, for instance, is the advice "to seal up in it [the pulp-canal] some of the essential oils." To seal up means the retention of all the gases of decomposition, necessarily forcing the septic matter through the foramen, increasing the inflammation of the pericementum. Again, "Lumps of ice wrapped in muslin may be placed between the lip and the tooth." Will the author kindly explain the effect of cold upon the peripheral blood-vessels in his next edition? Will these not be contracted and the contained blood be forced to the focus of inflammation, thus increasing the hyperæmia of the part? The student following the author's advice would certainly have quickly an abscess to deal with.

The rather dogmatic assertion that "the so-called 'green stain' of childhood is wholly superficial and has no pathological signification" needs qualification. Those who accept Williams's dictum, endorsed by Black, that the gelatinous plaques upon teeth are a cause of decay, can hardly be consistent and refuse to believe that the organisms of green stain possess the same quality. It is true that the special organism producing this has not been isolated, but the clinical fact remains that the enamel of all teeth so affected, even in later life, are more or less eroded, and in children the destruction is often serious. It is the opinion of some investigators that this destruction precedes the coloration. This has yet to be proved.

The reviewer never approaches the question of treatment of pyorrhœa alveolaris by writers in general, that he is not struck with the therapeutic limitations of the dental profession, and the author furnishes no exception to the rule. As far as he goes, the removal of "serumal tartar," the treatment of the pockets by acids, and the use of a "weak solution of chloride of zinc" as a stimulating astringent constitute the principal treatment. This, as a whole, is to the reviewer unscientific, as it is not based on the conditions present. Whatever discordant notes may be heard upon the etiology of this disease, there should be but one opinion,—that, properly managed, it can be cured, the exception being that which the reviewer designates as senile pyorrhœa; and this he holds to be practically incurable, for reasons that space will not permit him to give. The fact that similar conditions may cause a recurrence of this disease is admitted, but the physician who has given a clean bill of health to a patient recovering from typhoid fever would not say to him, You are immune from further attacks. The constant statement that pyorrhœa alveolaris is not cured because it returns, is an absurd position to take.

Chapter XLVII. is curious in that it seems to contravene all known conceptions of what constitutes secondary dentine, pulp nodules, and calcifications. The author starts out with this remarkable sentence: "These, although different manifestations, are parts of the same process. . . . All of these products have the general structure of dentine, although it may be considerably modified." Space or time is not afforded to enter upon an examination of this statement, but, as the reviewer understands it, this is entirely at variance with recognized histological facts, and, therefore, it was incumbent upon the author to give some reason for his opinion other than the single illustration which, while it bears out his conclusion, is evidently a true picture of secondary dentine, and not that of a pulp-stone as generally understood.

The chapter on syphilis is well prepared and equally well illustrated. It is, perhaps, one of the most satisfactory in the book.

The author is hardly excusable in omitting, in his otherwise valuable chapter on neuralgias, or in that on dentition, some allusion to the lesions resulting from impacted teeth. An entire chapter might profitably have been devoted to the reflexes resulting from these malpositions.

The consideration of the book cannot be extended farther. There is much that is valuable in it, but much that should have been corrected or entirely eliminated. The reviewer is aware of the fact that the preparation of a book on dental pathology involves greater difficulty, and is more likely to arouse adverse criticism, than any other subject connected with dental practice. It is the youngest of the specialties, and, as a result, methods of practice are not well established. While this is true, certain fixed principles of general pathology are understood and should be made to govern special practice.

The book is given with the usual care to detail of the S. S. White Dental Manufacturing Company.

